# The effectiveness of e-learning in patient education delivered to patients with rheumatoid arthritis: The WebRA study—protocol for a pragmatic randomised controlled trial

**DOI:** 10.1186/s41927-021-00226-y

**Published:** 2021-12-20

**Authors:** Line Raunsbæk Knudsen, Kirsten Lomborg, Mwidimi Ndosi, Ellen-Margrethe Hauge, Annette de Thurah

**Affiliations:** 1 Department of Rheumatology, Aarhus University Hospital, Palle Juul Jensens Boulevard 99, 8200 Aarhus, Denmark; 2 Department of Clinical Medicine, Aarhus University, Aarhus, Denmark; 3 Steno Diabetes Center Copenhagen, Gentofte, Denmark; 4 Department of Clinical Medicine, University of Copenhagen, Copenhagen, Denmark; 5 School of Health and Social Wellbeing, University of the West of England, Bristol, UK; 6 University Hospitals Bristol, Bristol, England

**Keywords:** Rheumatoid arthritis, Patient education, Web-based patient education, e-learning, Self-management, Randomised controlled trial

## Abstract

**Background:**

Patient education is integral to the treatment and care of patients with rheumatoid arthritis. Change is taking place in the organisation of healthcare systems because of a demographic shift towards ageing populations, an increasing use of technology and advancements in digital technologies, allowing for new interventions. This study will aim to evaluate the effectiveness of a newly developed e-learning patient education programme based on self-management that targets patients with rheumatoid arthritis.

**Methods:**

A pragmatic multi-centre randomised controlled trial is planned. We intend to recruit approximately 200 patients with a new diagnosis (< 3 months) of rheumatoid arthritis. Participants will be randomised 1:1 to web-based patient education delivered through an e-learning programme at home or standard face-to-face patient education provided at the hospital. The primary outcome is self-efficacy. Secondary outcomes are improved knowledge of rheumatoid arthritis, adherence to medication, health literacy level and quality of life. Outcomes will be measured at baseline and follow-up occurring 1, 3, 6 and 12 months after enrolment. Furthermore, data on healthcare utilisation and utilisation of the e-learning programme will be assessed at the 12-month follow-up. Statistical analysis, including differences between groups, will be evaluated using the chi-square and Kruskal–Wallis tests. Statistical analysis will follow the intention-to-treat principle, and analysis of variance will be used to evaluate the within- and between-groups differences testing the hypothesis of the ‘superiority’ of web-based patient education over standard face-to-face education provided at the hospital. Per protocol analysis will be used to assess the impact of missing data. Enrolment started in February 2021 and will end in June 2022.

**Discussion:**

The study is expected to contribute to the evidence on the effectiveness of web-based patient education within rheumatic diseases. If the e-learning programme is effective, it will be incorporated into existing services to improve the self-management of patients with rheumatoid arthritis. Further, this mode of providing patient education may impact the organisation of health care for both rheumatic diseases and other chronic diseases by offering different modes of delivering patient education based on the needs and preferences of patients.

*Trial registration*: ClinicalTrials.gov identifier NCT04669340. Registered on November 27, 2020. https://www.clinicaltrials.gov/ct2/show/NCT04669340?term=e-learning&cond=Rheumatoid+Arthritis&draw=2&rank=1. See Additional file 1 for detailed information on the dataset according to the World Health Organization Trial Registration Data Set.

**Supplementary Information:**

The online version contains supplementary material available at 10.1186/s41927-021-00226-y.

## Background

Patient education with the aim of supporting patients to self-manage their disease is considered an important part of the treatment and care offered to patients with rheumatoid arthritis (RA) [1–3]. RA is a chronic autoimmune inflammatory disease affecting the joints and organs [4, 5]; it has a worldwide prevalence of approximately 5 per 1000 people [6]. Pain and swelling of the joints and fatigue are common symptoms that may reduce physical function and affect quality of life (QoL) [6]. Furthermore, a report from the World Health Organization (WHO) highlights the strong relationships between painful musculoskeletal conditions and reduced physical activity, functional capacity and well-being [7]. Patient education enables patients to self-manage their illness and cope with their condition, thereby maintaining a higher QoL [1, 3].

The management and treatment of RA has improved significantly over the last 2 decades [2, 6]. Despite rising prevalence because of demographic development, i.e. the ageing population [7–10], the prognosis and outcomes of patients with RA have generally improved. However, this places new demands on the healthcare system and calls for alternative solutions to conventional models of care. The present advancements in digital technologies allow for the delivery of new interventions, including patient education delivered in combinations of text, images, audio, video, animations and interactive features to enhance access and understanding [11].

Nurses play an important role in supporting patients to participate actively in the treatment of their chronic conditions and in achieving self-management skills [12]. The Stanford Arthritis Self-Management Programme (ASMP) [13–15] is built on social cognitive theory [16] and is implemented for many chronic diseases [13–15]. According to the ASMP, self-management means having or being able to acquire the skills and resources necessary to best accommodate the chronic disease and its consequences [16]. Self-efficacy, that is, the confidence a person feels about performing a particular activity, is considered a pre-condition of self-management [17]. It has been shown that self-management education can lead to behaviour changes for patients with chronic diseases, and thus, improve outcomes in the forms of increased self-efficacy and treatment adherence, higher self-rated health, increased activity and decreased depression and anxiety [18].

Educational needs vary among individuals and can change throughout the course of a disease [1]. For this reason, it is recommended for patient education on RA to be individually tailored and cover several aspects, for example, knowledge of the disease and treatment, non-pharmacological treatment, pain control, self-help methods, activity regulation, physical exercise and emotional issues [1]. At the minimum, patient education must be offered at the time of diagnosis and when pharmacological treatment changes [1]. Patient education can be provided face-to-face or on the web, supplemented by phone calls and written or multimedia material [1].

Only a few randomised controlled trials (RCTs) have been conducted in the area of web-based patient education targeting people with RA. However, within both rheumatology (RA, fibromyalgia, low back pain) and other chronic diseases, the evidence points towards improvement of self-efficacy, knowledge, psychological distress and QoL in studies evaluating web-based self-management tools [19–23].

Evidence shows that so-called ‘entertainment education’ can improve self-efficacy by merging educational content with entertainment messages to increase knowledge, create favourable attitudes and change behaviour [24–27]. The main elements are narratives, stories and messages, combined with facts disseminated in an entertaining manner [24, 25].

As we write this protocol, approximately 90% of families in Europe have access to the internet [28]. However, there are variations in computer skills [28], and the use of health-related digital sources is lower among people with low educational levels [29]. A systematic review of 34 studies looking at the meaning of health literacy identified three key elements: (1) knowledge of health, healthcare and health systems; (2) processing and using information in various formats in relation to health and health care; and (3) the ability to maintain health through self-management and working in partnerships with health providers [30]. Health literacy is the degree to which individuals have the capacity to obtain, process and understand the basic health information and services needed to make appropriate health decisions [31]. We presume that health literacy skills may vary; therefore, health literacy principles must be considered when designing modern health information and services, as this may strengthen the usability for a broader audience [32].

Patient education based on digital technology may have several advantages. First, a web-based patient education programme can be accessed repeatedly, which can be favourable to the comprehension of the provided information and as needs changes; furthermore, repetition of a performance increases a person’s self-efficacy [17]. Second, since both auditory and visual channels are used, the integration of words and images in web-based programmes can promote deeper understanding and learning [33]. Moreover, digital tools provide flexibility and accessibility [11], and it may be beneficial for patients to access the programme at their time of convenience in familiar surroundings and possibly with relatives. Possible disadvantages of web-based patient education may be related to the absence of face-to-face contact with healthcare professionals who can facilitate an individual approach through a conversation. Moreover, healthcare professionals may encourage and motivate patients through relationships and face-to-face communication.

We have developed an e-learning programme, targeting patients with RA. The development draws on elements of the cognitive theory of multimedia learning [33], entertainment education [24, 25] and didactics to accommodate different ways of learning and levels of health literacy. It is divided into three learning modules: (1) knowledge of RA, the typical disease course, prognosis and medication; (2) additional information on RA, treatment possibilities and examinations; and (3) daily living with RA, for example, coping with emotions, pain, fatigue, activity, work and education. The programme offers a combination of animations, videos with patients and health professionals, graphics, podcasts, written text, speech and tests.

This paper reports the study protocol for an RCT evaluating the effectiveness of the described e-learning programme in improving patients’ self-management of RA. We hypothesise that the e-learning patient education programme will be superior to standard face-to-face patient education in improving the primary outcome, which is self-efficacy.

## Methods/design

**Fig. 1:**
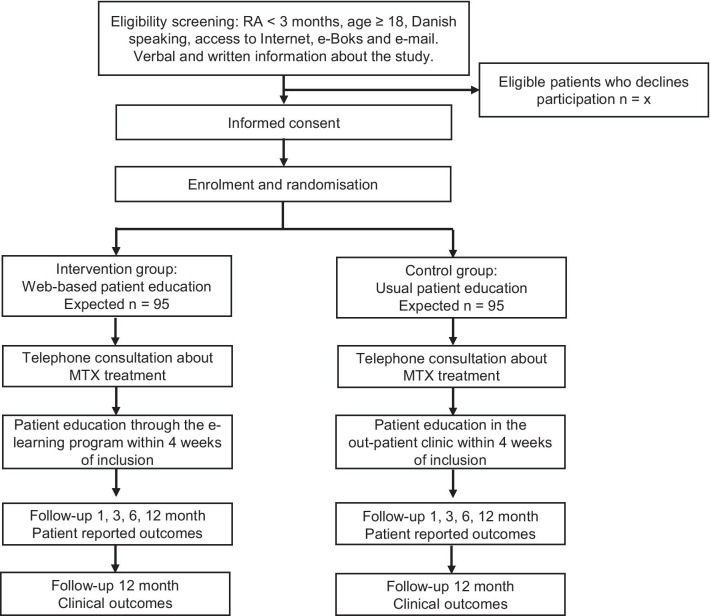
Flowchart showing steps from inclusion to data collection

The WebRA study is a pragmatic multi-centre RCT with two arms. In the study, participants will be randomised 1:1 to either the intervention group, with web-based patient education delivered through an e-learning programme provided at home, or control group, with standard face-to-face patient education provided at the outpatient clinic by a nurse. Enrolment started in February 2021 and is expected to be completed in June 2022. Follow-up and assessment of outcomes will be carried out 1, 3, 6 and 12 months after enrolment. Figure 1 presents the steps from enrolment to follow-up. This RCT will follow the Consolidated Standards of Reporting Trials (CONSORT) 2010 guideline [34] and the protocol follows the SPIRIT (Standard Protocol Items: Recommendations for Interventional Trials) guidelines and checklist [35].

### Study population and setting

Patients will be considered eligible for the study if they have been diagnosed with RA according to the American College of Rheumatology/European League Against Rheumatism 2010 (ACR/EULAR 2010) criteria [4] within the past 3 months. Patients will receive written and verbal information about the study and their rights as participants in a clinical trial by a study nurse and will be included if they fulfil the inclusion criteria and have signed informed consent. The study will be carried out at five rheumatology clinics in Denmark, which are as follows: Aarhus University Hospital, Aalborg University Hospital, Odense University Hospital, Randers Regional Hospital and Horsens Regional Hospital. Patients will be recruited in connection with an outpatient visit.

### Inclusion and exclusion criteria

The inclusion criteria are as follows:

- RA diagnosed according to the ACR/EULAR 2010 criteria within the past 3 months.
- Adults ≥ 18 years old.
- Ability to speak, read and understand Danish.
- Access to the internet at home.
- Access to e-Boks (secure online mailbox in the Danish public sector) and a private e-mail address.

Patients will be excluded if they already participated in formalised patient education when diagnosed with RA or if they withdraw consent.

### Medical treatment and standard care

According to guidelines, patients with RA are treated with disease-modifying anti-rheumatic drugs (DMARDs) based on disease activity, safety issues and individual patient factors [36]. Methotrexate (MTX) is the first drug of choice and will be escalated until treat-to-target, that is, remission or low disease activity [36]. Patient information on treatment, including treatment options, side effects and risk factors, should be provided throughout the course of disease and at medical treatment change [1, 2]. Thus, participants will receive written and verbal information about initiated medical treatment throughout the study period. Furthermore, information on symptoms and disease signs will be given to ensure appropriate reactions in the case of a disease flare or an infection.

### REDCap—Research Electronic Data Capture tool

Study data are collected and managed using Research Electronic Data Capture (REDCap) hosted at Aarhus University [37, 38]. REDCap is a secure, web-based software platform designed to support data capture for research studies [37, 38]. Thus, all data collected from the medical records, interviews with patients and questionnaires related to the outcomes of the study will be collected, administered, stored and managed through REDCap.

### Enrolment and randomisation

A study nurse will enrol patients in the study at a scheduled visit in the outpatient clinic or by phone. At enrolment, the study nurse will obtain demographic data through patient interviews and baseline data on diagnosis and medical treatment. Disease activity by the time of diagnosis will be obtained via the medical records (Table 1). Next, participants will be randomised using REDCap. The randomisation allocation tables in REDCap were performed by a data manager at Aarhus University without clinical involvement in the study, and the allocation tables were stored in a file only accessible by the independent data manager. Participants will be randomised in a ratio of 1:1. Because of the nature of the intervention, blinding is not possible. The randomisation is stratified by study site (Aarhus, Aalborg, Odense, Randers or Horsens), gender and age (< 69 years/> 70 years). A study nurse will perform the randomisation electronically in REDCap, whereby allocation concealment will be ensured. Competitive enrolment will occur to reach the target sample size in the enrolment period. Furthermore, an estimation of expected participants at each site, based on the numbers of patients with RA referred to the hospitals in 2019, was performed.

**Table 1:**
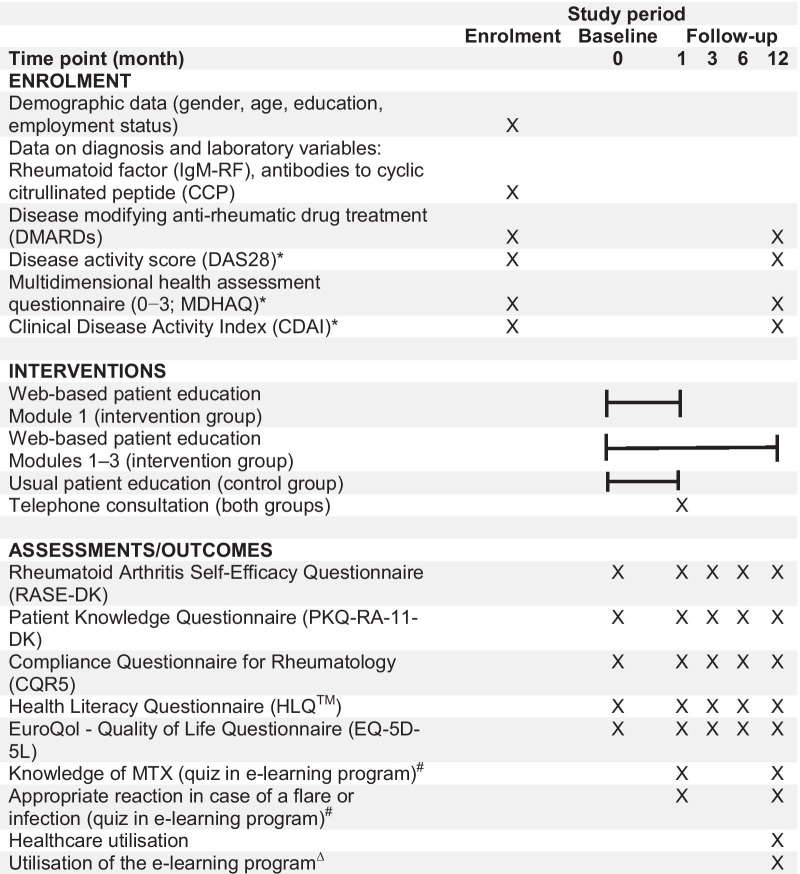
Time schedule and outcomes in the WebRA study

^*^At enrolment, scores for the DAS28, MDHAQ and CDAI are collected from the time of diagnosis, and at follow-up (12 months), scores are collected from a consultation in the outpatient clinic scheduled approximately 1 year after diagnosis

^#^‘Knowledge of MTX’ and ‘How to react to symptoms and when to contact health professionals’ are quizzes integrated into the e-learning program. Thus, these will be administered solely to participants in the intervention group

^∆^Data on the use of the e-learning patient education program are collected from the Learning Management System of the e-learning program

### Interventions

At enrolment, a study nurse briefly repeats information on the management of disease flares, infections and side effects for all participants. Moreover, a phone call is scheduled approximately 3 weeks after MTX initiation to ensure proper management of the medicine and to support treatment adherence [39]. All participants have access to telephone contact with the clinic as usual to ensure timely access to care [12].

#### Intervention group: web-based patient education

In the intervention group, participants will have access to the e-learning programme through a link sent to them personally by secure mail via e-Boks. They will be asked to accomplish module 1 of the programme within 4 weeks after enrolment and a reminder will be processed 4 days before deadline. The duration of the module is approximately 45 min, but users can pause and return whenever they want. Participants will be encouraged to go through the programme as many times as necessary and to involve family and relatives if they like. When module 1 is completed, modules 2 and 3 can be accessed and the e-learning programme can be used as needed throughout the study period.

In module 1, patients complete two tests to examine their knowledge of MTX and appropriate actions taken by the patient in case of a disease flare or infection. One ‘stop’ question regarding the dosage of MTX is formulated, and in case of a wrong answer to this specific question, the project manager (LRK) will contact the treating clinic to ensure contact with the patient (Additional file 1).

#### Control group: usual patient education

In the control group, participants will receive standard face-to-face patient education from a nurse in the outpatient clinic within 4 weeks after enrolment. The duration of this visit is approximately 1 h, and relatives can take part in the conversation. This session will cover the following topics: knowledge of RA, the typical disease course, prognosis, medication and daily life with RA. The nurse will follow a guideline to ensure a certain degree of consistency regarding the content of the patient education; however, the information will be individually adjusted and based on the patients' needs.

## Outcomes

The effect of patient education delivered through e-learning and usual care in improving patients’ self-management of RA will be evaluated via the three following aspects: patient-reported outcomes, healthcare utilisation and utilisation of the e-learning programme. Participants will respond to questionnaires of patient-reported outcomes at baseline and after 1, 3, 6 and 12 months of follow-up. The questionnaires will be administered electronic through REDCap [37, 38], and if not answered, a reminder will be processed after 7 and 14 days. Variables representing the demographic, clinical, treatment and outcome data collected in the study are listed in Table 1.

### Primary outcome

The primary outcome is self-efficacy, measured by a disease-specific Rheumatoid Arthritis Self-Efficacy (RASE) questionnaire [40]. The Danish version of RASE showed specificity and sensitivity to detect self-efficacy in alignment with the original British version [40]. Furthermore, RASE has been used as a primary outcome in an RCT investigating the impact of different types of outpatient care on self-efficacy [41]. RASE covers beliefs about the ability to perform tasks related to eight dimensions of self-management (relaxation, relationships, function, leisure activities, exercise, sleep, medication, fatigue) [42]. The items have five response options (1: strongly disagree, 2: disagree, 3: neither disagree nor agree, 4: agree, 5: strongly agree) and a score range of 28–140, with higher scores indicating greater self-efficacy [40, 42].

### Secondary outcomes

The secondary outcomes are knowledge of RA [43, 44], adherence to medication [45], health literacy level [45, 46] and QoL [46, 47].

### Knowledge of RA

Knowledge of RA will be measured by a Patient Knowledge Questionnaire (PKQ) for patients with RA. This questionnaire is based on the original PKQ for RA by Hill et al. [43] and the PKQ for early arthritis by Hennel et al. [44] which was developed to assess knowledge of RA following patient education. As the original PKQ was developed before the current management of RA using the treat-to-target approach, the target being remission, we have updated, translated and adapted the PKQ into Danish. The adapted and validated Danish version has good face validity, reflecting the content of the e-learning program, as well as current treatment guidelines and targets. The PKQ translation and validation study is in preparation for publication elsewhere.

The adapted questionnaire consists of 11 multi-choice questions covering disease aetiology, signs and symptoms, drug therapy and monitoring, joint protection and exercise, and energy conversation [44]. In the adapted Danish version, each question has one, two or three correct answers, and inspired by the original version, the total score ranges from 0 to 11; higher scores indicate a higher level of knowledge [43, 44]. The English versions of the instrument has been shown to be reliable and to have face validity [43, 44], and recently, the PKQ was used in two studies evaluating multimedia educational tools for patients with RA [26, 27].

### Adherence to medication

We chose the Compliance Questionnaire for Rheumatology (CQR5) as a measure for drug adherence [45]. The CQR5 is a reduced version of the original Compliance Questionnaire for Rheumatology with 19 items (CQR19) [48, 49]. The validation of the CQR5 has been shown to maintain the reliability and validity assessed in CQR19 while improving the utility in clinical practice by offering a quicker and easier tool to manage [45]. It consists of five questions derived from the original CQR19 covering reasons to adhere to medications. Answers to each question are given on a 4-point Likert scale ranging from *‘Definitely don’t agree’* (1) to *‘Definitely agree’* (4), with lower scores indicating lower levels of adherence [45]. A previous factor analysis showed that the CQR5 correctly predicted group membership measured by the full CQR19 for 88.5% of the cases, with a sensitivity to predict low adherence of 69% and specificity of predicting high adherence of 97% [45]. The original CQR19 has been translated and validated into Danish in connection with a study investigating adherence to MTX [50]. Prior to the present study, we extracted the five questions corresponding to the CQR5 from the Danish CQR19 and evaluated the reliability of the Danish CQR5. This reliability study is in preparation.

### Health literacy

Health literacy will be measured using the Health Literacy Questionnaire (HLQ™) [51, 52]. The HLQ has nine sub-scales covering different aspects of health literacy, and each can be used independently [51, 52]. Sub-scales 2 (*‘Having sufficient information to manage my health’*)*,* 4 (*‘Social support for health’*)*,* 6 (‘*Ability to actively engage with healthcare providers’*) and 9 (*‘Understand health information well enough to know what to do’*) were chosen for this study. Scales 2 and 4 have four response categories (strongly disagree, disagree, agree and strongly agree), and scales 6 and 9 have five response categories (cannot do, very difficult, quite difficult, quite easy and very easy) [52]. The Danish version has shown strong construct and content validity and high composite reliability [52].

### QoL

QoL will be measured using the EuroQol (EQ-5D-5L) questionnaire [46, 47]. The EQ-5D-5L has the five following dimensions: mobility, self-care, usual activities, pain/discomfort and anxiety/depression [47]. It has the five following response categories, which reflect five levels of severity: ‘*no problems*’, ‘*slight problems*’, ‘*moderate problems*’, ‘*severe problems*’ and ‘*extreme problems*’. In addition, respondents rate their overall health for the day using a visual analogue scale (VAS) [47]. The instrument has been widely tested and used in both general and patient populations, including the population with RA [47, 53].

### Clinical outcomes

The clinical outcomes of RA will be retrieved from patients’ medical records. Based on the ACR/EULAR recommendations on how to report disease activity in clinical trials, the Disease Activity Score based on the 28-joint count (DAS28) and the Clinical Disease Activity Index (CDAI) [54] will be used. DAS28 is a composite score consisting of the 28-joint count, laboratory variable and patient’s assessment of general health on 100 mm VAS [55, 56]. Scores range from 0 to 9.4 and disease activity is classified as remission, low, moderate or high [56]. The CDAI is a simple measure of disease activity of 28 joints without laboratory variables, integrating both the physician and the patient global assessment of disease activity [56]. Scores range from 0 to 76.0, and disease activity is classified as remission, low, moderate or high [56]. Functional status will be measured by the Multidimensional Health Assessment Questionnaire (MDHAQ) [57], which comprises 10 items related to activities of daily living. Each item has four response categories (without any difficulty, with some difficulty, with much difficulty, unable to do). Item scores range from 0 to 3; higher scores indicate worse function and greater physical disability [57].

### Healthcare utilisation

After completion of the follow-up period, data on the utilisation of healthcare in the study period will be retrieved from the medical record. This includes the number of contacts with the outpatient clinic, that is, telephone contacts with the rheumatology staff, as well as planned and acute visits. The focus for the contact or visit will be categorised within the following topics: flare, joint injection, medical treatment, medical investigations (e.g., bloods, magnetic resonance imaging [MRI] or X ray), information and guidance related to RA or self-administration of medicine.

### Utilisation of the e-learning program

By the end of the study, data on the use of the e-learning programme will be assessed. This includes the completion of modules, time spent, the number of logins to the program and the results of tests from the e-learning programme.

## Statistical methods

### Sample size estimation

Self-efficacy was the basis for the sample size estimation. An RCT on the effect of internet-based patient education through an online self-management program for RA among 93 patients found a mean self-efficacy score of 84.1 (standard deviation [SD] 16.3) in the intervention group receiving online education and 68.6 (SD 23.3) in the control group receiving standard patient education [19]. This equals a mean difference of 15.5 (standard error [SE]: 4.27). Based on the SE, we calculated the SD to 20.1. Based on this previous study, we wish to see at least a difference of 10% between the groups [19]. Given a statistical power of 90%, *p* value = 0.05, we will need a sample size of 80 patients in each group. To account for attrition and loss to follow-up, we intend to recruit 190 participants.

### Statistical analysis

Descriptive statistics will be used to describe and summarise the characteristics of the participants, that is, demographic data, disease-specific variables, treatment and covariates; the DAS28, MDHAQ and CDAI; and the allocation of participants to trial groups. Differences between groups for the primary and secondary outcome measures from baseline to the 12-month follow-up will be evaluated by the chi-square test for dichotomous variables and the Kruskal–Wallis test for continuous variables.

Differences within and between groups for the primary and secondary outcomes will be assessed using repeated measures modelling, that is, the one-way analysis of variance (ANOVA) test, including the *F* test. An additional post hoc test will be done to determine where the difference is. This will account for time, group and interaction effects. To enable intention-to-treat (ITT) analysis, missing data will be imputed using single imputation, that is, replacement of the missing value by the estimated population mean or median, depending on the distribution of data. In addition to the ITT analysis, a conservative complete case (per protocol [PP]) analysis will be conducted to assess the impact of the missing data. The PP analysis will be based on evaluation of all participants who completed the 12-month follow-up. Results from the ITT and PP analyses will be compared to assess the efficacy of the intervention. All analyses will be adjusted for baseline values. Summary statistics will be presented as means with standard deviation and 95% confidence interval for continuous variables with a normal distribution, numbers and percentages for dichotomous categories and logarithm transformed data as median with 95% confidence interval. STATA version 16.1 [58] will be used in all statistical analyses.

### Organisation and patient involvement

A steering group with collaborators from the participating sites, patient research partners and supervisors has been established. The group represents expertise in rheumatology, clinical epidemiology, qualitative research and the patient perspective. Furthermore, two international collaborators with expertise in patient education in inflammatory arthritis are involved. To ensure the progress of the project, the project leader, LRK, will provide close follow-up in all phases. Meetings with the steering group are planned twice a year and frequently contact between the project leader and study nurses will occur to ensure the completion of the study by ongoing evaluation and adjustment of procedures. At each participating site a lead investigator is identified and study nurses are affiliated the study, and are responsible for identification of eligible participants, recruitment, enrolment and data collection. LRK and AdT will also participate in the recruitment of participants and data collection. Lead investigators will be steering group members. A monthly newsletter is send to all sites with up-dates about the recruitment rate and data collection.

Two patient research partners are involved in the project and have contributed to the development of the e-learning program, discussions on the planning of the RCT and patient information material. Involving patient research partners in research projects is strongly recommended because they contribute experiential knowledge, and thus, promote research based on the needs of patients [59]. The patient research partners will also be involved in the discussion of the results and dissemination.

## Discussion

Patient education and guidance is needed by most patients when newly diagnosed because of their disease-related distress and the uncertainty of living with a new long-term condition. Evidence on the effect of web-based patient education and online modes of delivering patient education in RA is lacking; hence, there is a need for this study. If the intervention is shown to be effective, the intention is to further develop and provide a patient education intervention to support self-management in patients with RA. We intend to expand on modes of delivering patient education by offering different ways of learning, accommodating different needs, abilities and competences among patients. The target group of the present study is newly diagnosed patients; however, we expect the programme to be a supportive tool throughout the course of the disease because it offers a wide range of information related to RA, as well as guidance and inspiration towards various aspects of life with RA. Presumably, not all patients will benefit from web-based patient education. Even so, face-to-face patient education may be replaced by web-based patient education for some and a supplement for others. In addition, it may contribute to bridging the gap between the healthcare system and everyday life by being an ongoing accessible resource.

We believe there is methodological strength, as the effectiveness is being tested in a randomised controlled study design, with reduced risk of bias. An equally important strength is the study sample size and the involvement of several rheumatology clinics representing a wide group of patients, which will enhance the external validity because of recruitment from both university and regional hospital referral areas. We expect this study to be feasible and acceptable to both participants and health professionals, as a minimum resource from both parts is required.

A limitation of this study could be the risk of introducing a potential selection bias; this could arise because we can only include patients with access to the internet. However, in 2019, only 6% of families in Denmark lacked internet access [28]. Another concern could be patients’ willingness to participate because some may reject participation as a result of worries about technical issues. This could result in the exclusion of patients with low health literacy. The study could also be limited by loss to follow-up and lower response rates of the repeated follow-up outcome questionnaires. However, to accommodate this, we have accounted for a 15% dropout rate in the power calculation, and reminders on questionnaires will be sent twice.

After the RCT, the two following additional studies will be carried out: (1) a qualitative study exploring the patient perspective on web-based patient education and (2) an implementation study based on the non-adoption, abandonment, scale-up, spread and sustainability (NASSS) framework [60] to prepare this new technology for application in daily clinical practice. Overall, we expect the results to contribute new insights into the area of patient education within RA, and we think that the study will be useful to future healthcare organisations because it will offer different modes of patient education and allow for individual adaptations.

## Supplementary Information

**Additional file 1**. Data set according to the World Health Organization Trial Registration Data Set.

## Data Availability

Because of confidentiality concerns, the datasets generated and analysed during the current study will not be publicly available; however, they will be available from the corresponding author on reasonable request. The dissemination plan of the results will be directed to different stakeholders, including patients and the public, health professionals within rheumatology and scientific circles. Results will be published in an international peer-reviewed journal.

## Abbreviations

ASMP: Stanford Arthritis Self-Management Programme
ACR/EULAR 2010 criteria: American College of Rheumatology/European League Against Rheumatism 2010 criteria
ANOVA: Analysis of variance
CDAI: Clinical Disease Activity Index
CSM: Common sense model of health and illness
CQR: Compliance questionnaire for rheumatology
CONSORT: Consolidated Standards of Reporting Trials
DAS28: Disease Activity Score
HLQ: Health Literacy Questionnaire
ITT: Intention-to-treat
MTX: Methotrexate
MDHAQ: Multidimensional Health Assessment Questionnaire
NASSS: Non-adoption, abandonment, scale-up, spread and sustainability
PKQ: Patient Knowledge Questionnaire
PP: Per protocol
QoL: Quality of life
RA: Rheumatoid arthritis
RCT: Randomised controlled trial
REDCap: Research Electronic Data Capture
RASE: Rheumatoid Arthritis Self-Efficacy Questionnaire
SPIRIT: Standard Protocol Items: Recommendations for Interventional Trials
VAS: Visual analogue scale

## References

[1] Zangi HA, Ndosi M, Adams J, Andersen L, Bode C, Boström C (2015). EULAR recommendations for patient education for people with inflammatory arthritis. Ann Rheum Dis.

[2] Combe B, Landewe R, Daien CI, Hua C, Aletaha D, Álvaro-Gracia JM (2017). 2016 update of the EULAR recommendations for the management of early arthritis. Ann Rheum Dis.

[3] Nikiphorou E, Santos EJF, Marques A, Böhm P, Bijlsma JW, Daien CI, et al. 2021 EULAR recommendations for the implementation of self-management strategies in patients with inflammatory arthritis. Ann Rheum Dis. 2021. 10.1136/annrheumdis-2021-220249 PMC8458093 33962964

[4] Aletaha D, Neogi T, Silman AJ, Funovits J, Felson DT, Bingham CO (2010). 2010 Rheumatoid arthritis classification criteria: an American College of Rheumatology/European League Against Rheumatism collaborative initiative. Arthritis Rheum.

[5] Otón T, Carmona L (2019). The epidemiology of established rheumatoid arthritis. Best Pract Res Clin Rheumatol.

[6] Aletaha D, Smolen JS (2018). Diagnosis and management of rheumatoid arthritis: a review. JAMA.

[7] Briggs AM, Cross MJ, Hoy DG, Sànchez-Riera L, Blyth FM, Woolf AD (2016). Musculoskeletal health conditions represent a global threat to healthy aging: a report for the 2015 World Health Organization world report on ageing and health. Gerontologist.

[8] Cross M, Smith E, Hoy D, Carmona L, Wolfe F, Vos T (2014). The global burden of rheumatoid arthritis: estimates from the global burden of disease 2010 study. Ann Rheum Dis.

[9] Minichiello E, Semerano L, Boissier MC (2016). Time trends in the incidence, prevalence, and severity of rheumatoid arthritis: a systematic literature review. Joint Bone Spine.

[10] Safiri S, Kolahi AA, Hoy D, Smith E, Bettampadi D, Mansournia MA (2019). Global, regional and national burden of rheumatoid arthritis 1990–2017: a systematic analysis of the Global Burden of Disease study 2017. Ann Rheum Dis.

[11] Lopez-Olivo MA, Suarez-Almazor ME (2019). Digital patient education and decision aids. Rheum Dis Clin N Am.

[12] Bech B, Primdahl J, van Tubergen A, Voshaar M, Zangi HA, Barbosa L (2020). 2018 update of the EULAR recommendations for the role of the nurse in the management of chronic inflammatory arthritis. Ann Rheum Dis.

[13] Lorig KR, Holman H (2003). Self-management education: history, definition, outcomes, and mechanisms. Ann Behav Med.

[14] Lorig KR, Ritter P, Stewart AL, Sobel DS, Brown BW, Bandura A (2001). Chronic disease self-management program: 2-year health status and health care utilization outcomes. Med Care.

[15] Lorig KR, Sobel DS, Stewart AL, Brown BW, Bandura A, Ritter P (1999). Evidence suggesting that a chronic disease self-management program can improve health status while reducing hospitalization: a randomized trial. Med Care.

[16] Holman HR, Lorig K, Schwarzer R (1992). Perceived self-efficacy in self-management of chronic disease. Self-efficacy: thought control of action.

[17] Bandura A, Schwarzer R (1992). Exercise of personal agency through the self-efficacy mechanism. Self-efficacy: thought control of action.

[18] Brady TJ, Murphy L, O'Colmain BJ, Beauchesne D, Daniels B, Greenberg M (2013). A meta-analysis of health status, health behaviors, and health care utilization outcomes of the Chronic Disease Self-Management Program. Prev Chronic Dis.

[19] Shigaki CL, Smarr KL, Siva C, Ge B, Musser D, Johnson R (2013). RAHelp: an online intervention for individuals with rheumatoid arthritis. Arthritis Care Res (Hoboken).

[20] Lorig KR, Ritter PL, Laurent DD, Plant K (2008). The internet-based arthritis self-management program: a one-year randomized trial for patients with arthritis or fibromyalgia. Arthritis Rheum.

[21] Weymann N, Dirmaier J, von Wolff A, Kriston L, Härter M (2015). Effectiveness of a Web-based tailored interactive health communication application for patients with type 2 diabetes or chronic low back pain: randomized controlled trial. J Med Internet Res.

[22] Lorig K, Ritter PL, Laurent DD, Plant K, Green M, Jernigan VB (2010). Online diabetes self-management program: a randomized study. Diabetes Care.

[23] Armstrong AW, Kim RH, Idriss NZ, Larsen LN, Lio PA (2011). Online video improves clinical outcomes in adults with atopic dermatitis: a randomized controlled trial. J Am Acad Dermatol.

[24] Nariman HN (1993). Soap operas for social change; towards a methodology for entertainment-education television.

[25] Arvind S, Michael JC, Everett MR, Miguel S (2003). Entertainment-education and social change: history, research, and practice.

[26] Lopez-Olivo MA, Ingleshwar A, Volk RJ, Jibaja-Weiss M, Barbo A, Saag K (2018). Development and pilot testing of multimedia patient education tools for patients with knee osteoarthritis, osteoporosis, and rheumatoid arthritis. Arthritis Care Res (Hoboken).

[27] Lopez-Olivo MA, Lin H, Rizvi T, Barbo A, Ingleshwar A, des Bordes JKA, et al. Randomized Controlled Trial of Patient Education Tools for Patients with Rheumatoid Arthritis. Arthritis Care Res (Hoboken). 2020. 10.1002/acr.24362 PMC10521328 32583971

[28] Danmarks Statistik. IT-anvendelse i befolkningen 2019. 2020.

[29] ECDC ECFDP. A literature review on health informationseeking behaviour on the web: a health consumer and health professional perspective. 2011.

[30] Liu C, Wang D, Liu C, Jiang J, Wang X, Chen H (2020). What is the meaning of health literacy? A systematic review and qualitative synthesis. Fam Med Commun Health.

[31] Sørensen K, Van den Broucke S, Fullam J, Doyle G, Pelikan J, Slonska Z (2012). Health literacy and public health: a systematic review and integration of definitions and models. BMC Public Health.

[32] Brach C, Keller D, Hernandez LM, Baur C, Parker R, Dreyer B, Schyve P, Lemerise AJ, Schillinger D. Ten attributes of health literate health care organizations. 2012.

[33] Mayer RE (2014). The cambridge handbook of multimedia learning.

[34] Schulz KF, Altman DG, Moher D (2010). CONSORT 2010 statement: updated guidelines for reporting parallel group randomised trials. BMJ.

[35] Chan AW, Tetzlaff JM, Altman DG, Laupacis A, Gøtzsche PC, Krle AJK (2015). SPIRIT 2013 Statement: defining standard protocol items for clinical trials. Rev Panam Salud Publica.

[36] Smolen JS, Landewé RBM, Bijlsma JWJ, Burmester GR, Dougados M, Kerschbaumer A (2020). EULAR recommendations for the management of rheumatoid arthritis with synthetic and biological disease-modifying antirheumatic drugs: 2019 update. Ann Rheum Dis.

[37] Harris PA, Taylor R, Thielke R, Payne J, Gonzalez N, Conde JG (2009). Research electronic data capture (REDCap)–a metadata-driven methodology and workflow process for providing translational research informatics support. J Biomed Inform.

[38] Harris PA, Taylor R, Minor BL, Elliott V, Fernandez M, O'Neal L (2019). The REDCap consortium: building an international community of software platform partners. J Biomed Inform.

[39] Ritschl V, Stamm TA, Aletaha D, Bijlsma JWJ, Böhm P, Dragoi RG (2021). 2020 EULAR points to consider for the prevention, screening, assessment and management of non-adherence to treatment in people with rheumatic and musculoskeletal diseases for use in clinical practice. Ann Rheum Dis.

[40] Primdahl J, Wagner L, Hørslev-Petersen K (2010). Self-efficacy in rheumatoid arthritis: translation and test of validity, reliability and sensitivity of the Danish version of the Rheumatoid Arthritis Self-Efficacy Questionnaire (RASE). Musculoskelet Care.

[41] Primdahl J, Wagner L, Holst R, Hørslev-Petersen K (2012). The impact on self-efficacy of different types of follow-up care and disease status in patients with rheumatoid arthritis–a randomized trial. Patient Educ Couns.

[42] Brady TJ (2011). Measures of self-efficacy: Arthritis Self-Efficacy Scale (ASES), Arthritis Self-Efficacy Scale-8 Item (ASES-8), Children's Arthritis Self-Efficacy Scale (CASE), Chronic Disease Self-Efficacy Scale (CDSES), Parent's Arthritis Self-Efficacy Scale (PASE), and Rheumatoid Arthritis Self-Efficacy Scale (RASE). Arthritis Care Res (Hoboken).

[43] Hill J, Bird HA, Hopkins R, Lawton C, Wright V (1991). The development and use of Patient Knowledge Questionnaire in rheumatoid arthritis. Br J Rheumatol.

[44] Hennell SL, Brownsell C, Dawson JK (2004). Development, validation and use of a patient knowledge questionnaire (PKQ) for patients with early rheumatoid arthritis. Rheumatology (Oxford).

[45] Hughes LD, Done J, Young A (2013). A 5 item version of the Compliance Questionnaire for Rheumatology (CQR5) successfully identifies low adherence to DMARDs. BMC Musculoskelet Disord.

[46] Rabin R, de Charro F (2001). EQ-5D: a measure of health status from the EuroQol Group. Ann Med.

[47] Herdman M, Gudex C, Lloyd A, Janssen M, Kind P, Parkin D (2011). Development and preliminary testing of the new five-level version of EQ-5D (EQ-5D-5L). Qual Life Res.

[48] de Klerk E, van der Heijde D, van der Tempel H, van der Linden S (1999). Development of a questionnaire to investigate patient compliance with antirheumatic drug therapy. J Rheumatol.

[49] de Klerk E, van der Heijde D, Landewé R, van der Tempel H, van der Linden S (2003). The compliance-questionnaire-rheumatology compared with electronic medication event monitoring: a validation study. J Rheumatol.

[50] de Thurah A, Nørgaard M, Harder I, Stengaard-Pedersen K (2010). Compliance with methotrexate treatment in patients with rheumatoid arthritis: influence of patients' beliefs about the medicine. A prospective cohort study. Rheumatol Int.

[51] Osborne RH, Batterham RW, Elsworth GR, Hawkins M, Buchbinder R (2013). The grounded psychometric development and initial validation of the Health Literacy Questionnaire (HLQ). BMC Public Health.

[52] Maindal HT, Kayser L, Norgaard O, Bo A, Elsworth GR, Osborne RH (2016). Cultural adaptation and validation of the Health Literacy Questionnaire (HLQ): robust nine-dimension Danish language confirmatory factor model. Springerplus.

[53] Hurst NP, Kind P, Ruta D, Hunter M, Stubbings A (1997). Measuring health-related quality of life in rheumatoid arthritis: validity, responsiveness and reliability of EuroQol (EQ-5D). Br J Rheumatol.

[54] Aletaha D, Landewe R, Karonitsch T, Bathon J, Boers M, Bombardier C (2008). Reporting disease activity in clinical trials of patients with rheumatoid arthritis: EULAR/ACR collaborative recommendations. Ann Rheum Dis.

[55] Prevoo ML, van’t Hof MA, Kuper HH, van Leeuwen MA, van de Putte LB, van Riel PL (1995). Modified disease activity scores that include twenty-eight-joint counts. Development and validation in a prospective longitudinal study of patients with rheumatoid arthritis. Arthritis Rheum.

[56] Dougados M, Aletaha D, van Riel P (2007). Disease activity measures for rheumatoid arthritis. Clin Exp Rheumatol.

[57] Maska L, Anderson J, Michaud K (2011). Measures of functional status and quality of life in rheumatoid arthritis: Health Assessment Questionnaire Disability Index (HAQ), Modified Health Assessment Questionnaire (MHAQ), Multidimensional Health Assessment Questionnaire (MDHAQ), Health Assessment Questionnaire II (HAQ-II), Improved Health Assessment Questionnaire (Improved HAQ), and Rheumatoid Arthritis Quality of Life (RAQoL). Arthritis Care Res (Hoboken).

[58] 16.1 S. StataCorp. Stata Statistical Software: Release 16. College Station, TX: StataCorp LLC. 2019.

[59] de Wit MP, Berlo SE, Aanerud GJ, Aletaha D, Bijlsma JW, Croucher L (2011). European League Against Rheumatism recommendations for the inclusion of patient representatives in scientific projects. Ann Rheum Dis.

[60] Greenhalgh T, Wherton J, Papoutsi C, Lynch J, Hughes G, A’Court C (2017). Beyond adoption: a new framework for theorizing and evaluating nonadoption, abandonment, and challenges to the scale-up, spread, and sustainability of health and care technologies. J Med Internet Res.

