# Supplementary material for: The effectiveness of e-learning in patient education delivered to patients with rheumatoid arthritis: The WebRA study—protocol for a pragmatic randomised controlled trial
Source: BMC Rheumatol. 2021 Dec 20;5:57. doi: 10.1186/s41927-021-00226-y (PMC8686289; doi:10.1186/s41927-021-00226-y)
Supplement: Supplementary file 1 — Additional file 1. Data set according to the World Health Organization Trial Registration Data Set. [file 41927_2021_226_MOESM1_ESM.docx]

###### File I

###### Data set according to the World Health Organization Trial Registration Data Set

| **Data category** | **Information** |
| --- | --- |
| Primary registry and trial identifying number | ClinicalTrials.gov: NCT04669340 |
| Date of registration in primary registry | 27 November, 2020 |
| Secondary identifying numbers | N/A |
| Source of monetary or material support | TrygFonden (grant number: 149582) |
| Primary sponsor | Department of Rheumatology, Aarhus University Hospital, Denmark. Contact: Ulrik Tarp, MD, DMSc  Chief physician. Email: [ulritarp@rm.dk](mailto:ulritarp@rm.dk). Telephone + 45 40343243 |
| Secondary sponsors | N/A |
| Contact for public queries | Line Raunsbæk Knudsen, RN, MCN, PhD student.  E-mail: linknuds@rm.dk. Telephone: + 45 30375716 |
| Contact for scientific queries | Line Raunsbæk Knudsen, RN, MCN, PhD student.  E-mail: linknuds@rm.dk. Telephone: + 45 30375716 |
| Public title | WebRA, e-learning – know your rheumatoid arthritis |
| Scientific title | WebRA, e-learning in patient education to patients with rheumatoid arthritis – a pragmatic randomised controlled trial |
| Countries of recruitment | Denmark |
| Health conditions or problems studied | Web-based patient education |
| Interventions | Intervention: Web-based patient education delivered through an e-learning programme at home.  Control: Standard face-to-face patient education provided at the hospital. |
| Key inclusion and exclusion criteria | Ages eligible for study: > 18 years Sexes *eligible for study: both* Accepts healthy volunteers: no  Inclusion criteria: adult patients (> 18 years) newly diagnosed with rheumatoid arthritis (< 3 months). Participants should be able to speak, read and understand Danish and have access to the internet at home, access to e-Boks (secure online mailbox in the Danish public sector) and a private e-mail address.  Exclusion criteria: Participation in other formalized patient education. |
| Study type | Interventional  Allocation: randomised  Intervention model: parallel assignment  Masking: N/A  Primary purpose: effectiveness |
| Date of first enrolment | 1 February 2021 |
| Target sample size | 190 |
| Recruitment status | Recruiting |
| Primary outcomes | Self-efficacy |
| Key secondary outcomes | Knowledge of rheumatoid arthritis  Adherence to medication  Health literacy level  Quality of life |
